# Prognostic Implications of p16 Immunohistochemistry and Its Discordance with Transcriptionally Active HPV in Oropharyngeal Squamous Cell Carcinoma: A Taiwanese Cohort Study

**DOI:** 10.3390/medicina62071401

**Published:** 2026-07-20

**Authors:** Yun-An Chen, Chien-Kuan Lee, Jen-Fan Hang, Chien-Chih Chen

**Affiliations:** 1 Department of Pathology and Laboratory Medicine, Taichung Veterans General Hospital, Taichung 40705, Taiwan; 2 Department of Pathology, Kuang-Tien General Hospital, Shalu, Taichung 433, Taiwan; 3 Department of Pathology and Laboratory Medicine, Taipei Veterans General Hospital, Taipei 40705, Taiwan; 4 School of Medicine and Institute of Clinical Medicine, National Yang Ming Chiao Tung University, Taipei 112304, Taiwan; 5 Department of Radiation Oncology, Taichung Veterans General Hospital, Taichung 40705, Taiwan; 6 Department of Medical Imaging and Radiological Sciences, Central Taiwan University of Science and Technology, Taichung 40705, Taiwan

**Keywords:** oropharyngeal squamous cell carcinoma, p16 immunohistochemistry, human papillomavirus, prognosis, HPV discordance

## Abstract

*Background and Objectives*: Oropharyngeal squamous cell carcinoma (OPSCC) is a biologically heterogeneous disease. p16 immunohistochemistry is widely used as a surrogate marker for human papillomavirus (HPV)–associated OPSCC and plays a key role in prognostication and staging. However, p16 overexpression does not invariably reflect transcriptionally active HPV infection, and the clinical significance of p16–HPV discordance remains incompletely defined, particularly in populations with high exposure to traditional carcinogens. *Materials and Methods*: We conducted a retrospective cohort study of patients diagnosed with OPSCC between 2016 and 2020 at a tertiary referral center in Taiwan. p16 immunohistochemistry was performed in all cases, and transcriptionally active high-risk HPV was assessed by RNA in situ hybridization in p16-positive tumors. Clinicopathologic characteristics, carcinogen exposure profiles, and survival outcomes, including overall survival (OS) and disease-free survival (DFS), were analyzed according to p16 and HPV status. *Results*: Among 140 patients with OPSCC, 49 (35%) were p16-positive, of whom 44 (90%) demonstrated transcriptionally active HPV. Compared with p16-negative tumors, p16-positive OPSCC was significantly associated with lymphoepithelial organ involvement, non-keratinizing morphology, lower clinical stage, and less exposure to alcohol, betel nut, and tobacco. In Kaplan–Meier and univariable analyses, p16 positivity was associated with significantly improved OS and DFS. In contrast, p16-positive/HPV RNA-negative tumors exhibited clinicopathologic features more closely resembling p16-negative OPSCC, including greater keratinization and higher carcinogen exposure. *Conclusions*: p16-positive/HPV RNA-negative tumors showed clinicopathologic features suggestive of biological heterogeneity; however, the small number of discordant cases precludes definitive conclusions regarding survival differences. These findings support selective HPV RNA testing in p16-positive OPSCC with atypical clinicopathologic features, particularly in carcinogen-endemic populations.

## 1. Introduction

Oropharyngeal squamous cell carcinoma (OPSCC) comprises a biologically heterogeneous group of tumors with distinct etiologic pathways and clinical behaviors. In recent decades, human papillomavirus (HPV)-associated OPSCC has emerged as a unique disease entity characterized by younger age at diagnosis, limited exposure to traditional carcinogens, and a significantly more favorable prognosis compared with HPV-independent disease [1,2]. This biological distinction has been formally recognized in the 8th edition of the American Joint Committee on Cancer (AJCC) staging system, which introduced separate staging criteria for HPV-associated OPSCC [2].

Immunohistochemical overexpression of p16 has been widely adopted as a surrogate marker for transcriptionally active high-risk HPV infection in OPSCC, owing to its high sensitivity, technical simplicity, and reproducibility in routine pathology practice [3,4]. Consequently, p16 positivity is frequently used to define HPV-associated OPSCC in both clinical trials and daily practice, and is often assumed to confer the same prognostic advantage as biologically confirmed HPV-driven tumors [3,4].

Nevertheless, accumulating evidence indicates that p16 overexpression is not exclusively driven by HPV-mediated oncogenesis. Alternative molecular mechanisms, including disruption of the retinoblastoma (Rb) pathway independent of HPV, may also lead to p16 upregulation [5,6]. As a result, a subset of OPSCC demonstrates discordance between p16 immunohistochemistry and direct HPV detection, raising concerns regarding the reliability of p16 as a stand-alone prognostic marker [6,7,8]. Several studies have reported that p16-positive but HPV-independent OPSCC may exhibit clinicopathologic features and clinical outcomes more closely resembling HPV-independent disease, although results across cohorts remain inconsistent [6,7,8,9].

The prevalence and clinical significance of HPV-associated OPSCC show substantial geographic variation. In Western countries, HPV infection accounts for the majority of OPSCC cases, whereas in many Asian populations, including Taiwan, HPV prevalence is lower and exposure to traditional carcinogens such as alcohol consumption, betel nut chewing, and cigarette smoking remains highly prevalent [10,11,12,13,14]. These epidemiologic differences suggest that the prognostic implications of p16 expression and its concordance with transcriptionally active HPV may not be directly extrapolated from Western cohorts to Asian populations. From a clinical perspective, accurate classification of HPV-associated OPSCC is important for prognostic counseling, interpretation of AJCC staging, comparison across clinical studies, and selection of patients for biomarker-driven risk stratification. Although p16 immunohistochemistry is practical and widely available, reliance on p16 alone may overestimate HPV-driven tumor biology in selected patients, particularly in populations with substantial exposure to alcohol, betel nut, and tobacco. Therefore, clarifying the relationship between p16 expression, transcriptionally active HPV, morphology, carcinogen exposure, and survival outcomes may help refine diagnostic interpretation in routine practice.

In this context, RNA-based in situ hybridization (ISH) targeting high-risk HPV E6/E7 transcripts has emerged as a highly specific method for detecting transcriptionally active HPV in formalin-fixed paraffin-embedded tissues and is considered a robust reference standard for biologically relevant HPV infection [15,16]. Evaluation of p16 expression in conjunction with HPV RNA status may therefore provide more refined risk stratification in OPSCC, particularly in regions with mixed etiologic exposures. Recent guideline updates continue to support p16 immunohistochemistry as the primary initial test for HPV-associated OPSCC, while emphasizing that HPV-specific testing may be appropriate in selected scenarios, including equivocal p16 staining, discordance between p16 expression and morphology, non-tonsillar or non–base of tongue tumor sites, and settings with lower HPV prevalence [17].

In the present study, we evaluated a cohort of Taiwanese patients with OPSCC to examine the prognostic significance of p16 immunohistochemistry and to characterize the clinicopathologic features and survival outcomes of p16-positive tumors according to HPV RNA status in a carcinogen-endemic population.

## 2. Materials and Methods

### 2.1. Study Design and Patient Selection

This retrospective cohort study was conducted at Taichung Veterans General Hospital, a tertiary referral medical center in Taiwan. Patients diagnosed with OPSCC between 1 January 2016 and 31 December 2020 were consecutively identified from the institutional pathology database. The study protocol was approved by the Institutional Review Board of Taichung Veterans General Hospital (IRB No. CE21193B).

Patients were included if they had a histopathologic diagnosis of primary OPSCC and received definitive treatment at our institution. Exclusion criteria were: (1) a history of prior head and neck squamous cell carcinoma; (2) the presence of synchronous head and neck malignancies at diagnosis; (3) absence of adequate formalin-fixed, paraffin-embedded (FFPE) tumor tissue for ancillary studies; or (4) incomplete clinical or follow-up data.

Clinical information, including age, sex, tumor site, tumor–node–metastasis (TNM) stage, treatment modality, and follow-up status, was retrieved from electronic medical records. Tumors were staged according to the American Joint Committee on Cancer (AJCC) 8th edition staging system.

For anatomic classification, primary tumor sites were categorized as lymphoepithelial (LE) or non–lymphoepithelial (non-LE) sites. In this adult OPSCC cohort, LE sites primarily included the tonsils and base of tongue, whereas non-LE sites included the soft palate and posterior pharyngeal wall. This classification was used because HPV-associated OPSCC preferentially arises from lymphoepithelial-rich oropharyngeal sites. For tumors involving both LE and non-LE sites, classification was based on the dominant epicenter of tumor involvement as determined by clinical examination, imaging findings, and pathologic assessment. Cases without a clearly dominant site were categorized as multifocal.

### 2.2. Assessment of Carcinogen Exposure

Information regarding exposure to traditional carcinogens, including alcohol consumption, betel nut chewing, and cigarette smoking, was obtained from documented clinical history in the medical records at diagnosis. Smoking history was further quantified in pack-years, and heavy smoking was defined as a cumulative exposure of more than 10 pack-years, a threshold commonly used in HPV-associated OPSCC risk stratification literature [18].

### 2.3. p16 Immunohistochemistry

p16 immunohistochemistry (IHC) was performed on 4-μm sections cut from FFPE tumor blocks using an automated staining platform (Ventana BenchMark XT; Ventana Medical Systems, Tucson, AZ, USA) with a monoclonal anti-p16 antibody (clone E6H4), following the manufacturer’s protocol.

Cases were considered p16-positive when ≥70% of tumor cells demonstrated moderate to strong nuclear and cytoplasmic staining, consistent with current guideline recommendations. The percentage of positive tumor cells, staining intensity, and staining distribution were assessed on representative tumor sections. Equivocal p16 staining patterns, including 50–70% tumor cell staining or extensive but weak staining, were specifically reviewed and adjudicated by consensus. Tumors were classified as p16-positive only when they met the predefined criterion of ≥70% tumor cells showing moderate to strong nuclear and cytoplasmic staining.

### 2.4. HPV RNA In Situ Hybridization

For p16-positive tumors, transcriptionally active high-risk human papillomavirus (HPV) infection was evaluated using RNA ISH. RNAscope^®^ technology (Advanced Cell Diagnostics, Newark, CA, USA) was applied using probes targeting E6/E7 transcripts of 18 high-risk HPV genotypes (HPV-HR18), according to the manufacturer’s instructions.

HPV-associated squamous cell carcinoma tissue served as an external positive control, while stromal and inflammatory cells within each specimen served as internal negative controls. Tumors showing distinct punctate nuclear and/or cytoplasmic signals in tumor cells were classified as HPV RNA-positive, whereas cases lacking specific tumor-cell signals were classified as HPV RNA-negative. HPV RNA status was analyzed as a binary variable in this study.

In this study, transcriptionally active HPV was operationally defined as HPV RNA ISH positivity based on the detection of E6/E7 transcripts from the high-risk HPV genotypes included in the RNAscope^®^ HPV-HR18 assay. HPV RNA ISH was performed in p16-positive tumors because p16 immunohistochemistry is commonly used as the initial screening test in routine diagnostic workflows, and the primary aim of this study was to evaluate p16–HPV discordance among p16-positive tumors.

### 2.5. Histopathologic Evaluation

All hematoxylin and eosin–stained slides and ancillary studies were independently reviewed by two pathologists who were blinded to clinical outcomes. Discrepancies were resolved by consensus review.

Tumor keratinization was semi-quantitatively categorized into three groups based on the proportion of keratinizing tumor cells: non-keratinizing (0–10%), non-keratinizing with maturation (10–50%), and keratinizing (>50%). The presence of associated high-grade epithelial dysplasia in adjacent mucosa was recorded using a binary grading system.

### 2.6. Survival Outcomes

Overall survival (OS) was defined as the interval from the date of diagnosis to death from any cause or last follow-up. Disease-free survival (DFS) was defined as the interval from diagnosis to disease recurrence, death, or last follow-up, whichever occurred first. Patients with follow-up shorter than six months were excluded from survival analyses to reduce bias from inadequate follow-up and early censoring.

### 2.7. Statistical Analysis

Categorical variables were compared using the chi-square test or Fisher’s exact test, as appropriate. Continuous variables were analyzed using the Kruskal–Wallis test. Survival curves were generated using the Kaplan–Meier method and compared with the log-rank test.

Univariable analyses of prognostic factors for OS and DFS were performed using Cox proportional hazards regression models. Hazard ratios (HRs) and 95% confidence intervals (CIs) were calculated. A two-tailed *p* value of <0.05 was considered statistically significant. All statistical analyses were conducted using MedCalc Statistical Software, version 20.109 (MedCalc Software Ltd., Ostend, Belgium).

Variables considered clinically relevant and/or significant in univariable analysis were evaluated in multivariable Cox proportional hazards models. To avoid model overfitting and collinearity, clinical stage was included instead of separate T and N classifications. Variables with complete separation were not included in the primary multivariable OS model.

### 2.8. Use of Generative AI and AI-Assisted Technologies

Generative AI and AI-assisted technologies were used only to improve language readability, grammar, and manuscript clarity after the initial drafting of the manuscript. ChatGPT (OpenAI, GPT-5.5 Thinking, accessed 15 July 2026) was used for language editing support. No AI-assisted tools were used for study design, data collection, pathological interpretation, statistical analysis, result interpretation, figure generation, or reference selection. All AI-assisted text was reviewed, edited, and approved by the authors, who take full responsibility for the accuracy and integrity of the manuscript.

## 3. Results

### 3.1. Patient Characteristics

A total of 140 patients with OPSCC were included in this study. The mean age at diagnosis was 57 years (range, 35–90 years), with a marked male predominance (male-to-female ratio, 7.75:1). The mean follow-up duration for the entire cohort was 35.6 months. Among all cases, 49 patients (35%) were classified as p16-positive OPSCC.

**Figure 1:**
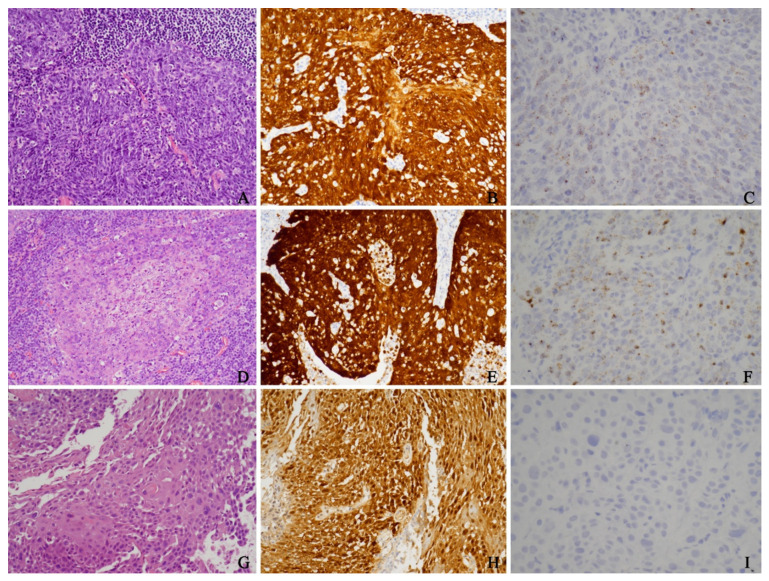
Representative histopathologic features, p16 immunohistochemistry, and HPV RNA in situ hybridization in oropharyngeal squamous cell carcinoma. (**A**–**C**) A representative non-keratinizing squamous cell carcinoma arising in a lymphoepithelial organ, showing sheets of tumor cells with prominent lymphoid stroma on hematoxylin and eosin staining ((**A**), ×100). Diffuse and strong nuclear and cytoplasmic p16 immunoreactivity is demonstrated ((**B**), ×100), accompanied by abundant punctate nuclear and granular cytoplasmic signals on HPV RNA in situ hybridization (ISH), consistent with transcriptionally active HPV infection ((**C**), ×200). (**D**–**F**) A non-keratinizing squamous cell carcinoma with focal maturation, exhibiting a reverse maturation pattern ((**D**), ×100). Tumor cells show diffuse strong p16 expression ((**E**), ×100) and high-level HPV RNA ISH signals ((**F**), ×200). (**G**–**I**) A representative p16-positive but HPV RNA–negative tumor, characterized by marked keratinization on hematoxylin and eosin staining ((**G**), ×100). Moderate to strong nuclear and cytoplasmic p16 expression is present ((**H**), ×100), whereas HPV RNA ISH shows no specific detectable signal ((**I**), ×200).

Within the p16-positive subgroup, transcriptionally active high-risk HPV was detected by RNA in situ hybridization (ISH) in 44 cases (90%), whereas 5 cases (10%) were HPV RNA-negative. In this study, HPV RNA-negative tumors were defined as tumors lacking detectable transcriptionally active HPV by RNA ISH. Representative histopathologic features, p16 immunohistochemical staining patterns, and HPV RNA ISH signals are illustrated in Figure 1.

### 3.2. Comparison Between p16-Positive and p16-Negative OPSCC

Clinicopathologic characteristics of p16-positive and p16-negative OPSCC are summarized in Table 1. Compared with p16-negative tumors, p16-positive OPSCC demonstrated a significantly lower proportion of male patients and was more frequently diagnosed at an early clinical stage (AJCC stage I–II). p16-positive tumors were also associated with lower T classification and showed a marked predilection for lymphoepithelial (LE) organ involvement.

Histopathologically, p16-positive OPSCC was characterized by a predominance of non-keratinizing morphology and a lower frequency of associated epithelial dysplasia. In contrast, p16-negative OPSCC exhibited significantly higher rates of keratinizing morphology.

With respect to environmental risk factors, patients with p16-positive OPSCC had significantly lower rates of alcohol consumption, betel nut chewing, and cigarette smoking, including heavy smoking defined as more than 10 pack-years, compared with those with p16-negative disease.

### 3.3. Comparison Between HPV RNA–Positive and HPV RNA–Negative Tumors Within the p16-Positive Group

Clinicopathologic comparisons between HPV RNA-positive and HPV RNA-negative tumors within the p16-positive group are detailed in Table 2. p16-positive/HPV RNA-negative tumors were less likely to arise from LE organs and more frequently originated from non-LE sites than HPV RNA-positive tumors.

Although both subgroups were predominantly diagnosed at early clinical stages, HPV RNA-negative tumors showed a significantly lower frequency of lymph node metastasis. Histopathologic evaluation revealed that HPV RNA-negative tumors exhibited significantly greater degrees of keratinization compared with HPV RNA-positive tumors.

In terms of carcinogen exposure, alcohol consumption, betel nut chewing, cigarette smoking, and heavy smoking were more prevalent among patients with p16-positive/HPV RNA-negative tumors than among those with HPV RNA-positive tumors. Notably, all five HPV RNA-negative cases had documented alcohol consumption and cigarette smoking, and four had a history of betel nut chewing. Given the small number of HPV RNA-negative cases, these subgroup comparisons should be interpreted as exploratory.

### 3.4. Survival Analysis

Seventeen patients with a follow-up duration of less than six months were excluded from survival analyses. Among the remaining 123 patients, the mean follow-up period was 65.5 months (range, 6.0–82.0 months). During follow-up, 30 patients died, including 2 patients in the p16-positive group and 28 patients in the p16-negative group. Disease recurrence occurred in 33 patients, of whom 2 were p16-positive and 31 were p16-negative.

**Figure 2:**
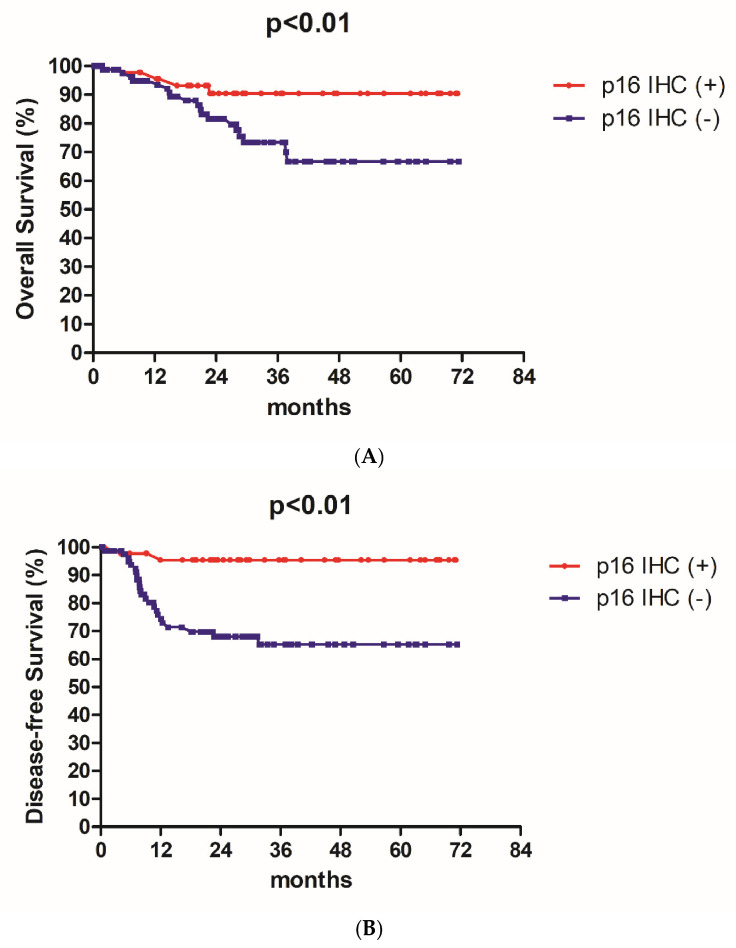
Kaplan–Meier survival analyses according to p16 status in oropharyngeal squamous cell carcinoma. (**A**) Overall survival and (**B**) disease-free survival stratified by p16 immunohistochemical status. Kaplan–Meier analysis showed significantly improved OS and DFS in patients with p16-positive OPSCC compared with those with p16-negative disease.

Kaplan–Meier survival analyses demonstrated significantly improved overall survival (OS) and disease-free survival (DFS) in patients with p16-positive OPSCC compared with those with p16-negative disease (Figure 2A,B).

### 3.5. Prognostic Factors

Univariable analyses of prognostic factors for OS and DFS are summarized in Table 3. p16 positivity and female sex were significantly associated with improved OS and DFS. Tumors arising from lymphoepithelial organs were also associated with favorable OS.

Conversely, advanced clinical stage, higher T classification, and keratinizing tumor morphology were significantly associated with poorer survival outcomes. Exposure to traditional carcinogens, particularly heavy cigarette smoking, was associated with worse OS and DFS.

Multivariable Cox proportional hazards analysis was performed to adjust for age, p16 status, clinical stage, keratinizing morphology, and betel nut chewing (Table 4). Advanced clinical stage remained independently associated with worse OS and DFS. In contrast, p16 status was not independently associated with OS or DFS after adjustment, suggesting that the prognostic effect of p16 may be partly explained by its association with stage and clinicopathologic features in this cohort.

Within the p16-positive subgroup, two deaths were observed, both in the HPV RNA-positive group. Disease recurrence occurred in two HPV RNA-positive cases, whereas no death or recurrence was observed in the HPV RNA-negative subgroup during the follow-up period. Owing to the limited number of events, formal statistical comparisons between the HPV RNA-positive and HPV RNA-negative subgroups were not performed.

## 4. Discussion

HPV-associated OPSCC is widely recognized as a biologically distinct disease entity with improved treatment response and survival compared with HPV-independent OPSCC [1,2]. Large multicenter studies, including the HNCIG-EPIC-OPC individual patient data analysis, have shown that p16/HPV concordance and discordance are associated with distinct clinical outcomes, supporting the clinical relevance of combined biomarker interpretation [19]. In our Taiwanese cohort, p16 positivity was associated with improved OS and DFS in univariable analyses. However, this association was attenuated after adjustment for clinical stage, keratinizing morphology, and carcinogen exposure, suggesting that the favorable prognosis of p16-positive OPSCC may be partly mediated by its association with favorable clinicopathologic features rather than by an entirely independent effect of p16 expression.

Our study also provides insight into the subset of p16-positive OPSCC lacking transcriptionally active HPV infection. In this cohort, 10% of p16-positive tumors were HPV RNA-negative by RNA ISH, a frequency comparable to that reported in several Western cohorts using RNA-based HPV detection methods [6,8,9]. These findings indicate that discordance between p16 overexpression and biologically active HPV infection is not uncommon and may occur across different geographic populations.

Importantly, the clinicopathologic features of p16-positive/HPV RNA–negative tumors in our cohort resembled those typically observed in HPV-independent OPSCC. These tumors demonstrated greater degrees of keratinization, preferential involvement of non–lymphoepithelial anatomic sites, and substantially higher exposure to traditional carcinogens, including alcohol consumption, betel nut chewing, and cigarette smoking. Such findings are consistent with prior studies suggesting that p16 overexpression can occur through HPV-independent molecular mechanisms, including disruption of the Rb pathway unrelated to viral oncogenesis [5,15]. Consequently, p16 expression alone may not uniformly reflect a biologically HPV-driven tumor phenotype.

The prognostic implications of p16–HPV discordance remain an area of ongoing debate. Earlier studies reported that p16-positive OPSCC conferred favorable outcomes regardless of HPV detection results [7], whereas more recent studies incorporating RNA-based HPV detection suggest that discordant tumors may show clinicopathologic and outcome profiles closer to HPV-independent disease [6,8,9,19,20,21]. Differences across studies may partly reflect variation in HPV detection methods. HPV DNA-based assays, including DNA in situ hybridization and PCR, detect viral DNA but do not necessarily confirm transcriptionally active viral oncogenesis. In contrast, RNA-based assays targeting E6/E7 transcripts more directly identify biologically active HPV infection and may reduce misclassification in discordant cases [17,22].

Because the p16-positive/HPV RNA-negative subgroup included only five cases and no documented recurrence, definitive conclusions regarding survival differences between concordant and discordant p16-positive tumors cannot be drawn. Nevertheless, the clinicopathologic characteristics observed in these tumors, including keratinizing morphology, non–lymphoepithelial location, and increased carcinogen exposure, suggest biological heterogeneity within p16-positive OPSCC. These findings should therefore be regarded as hypothesis-generating and require validation in larger cohorts.

Geographic and etiologic context is particularly important when interpreting the clinical significance of p16 expression. Recent Asian data, including a Chinese/Asian OPSCC cohort and a nationwide Chinese HNC study, support the possibility that HPV-associated disease differs by regional HPV prevalence, carcinogen exposure, tumor presentation, and survival impact [21,23]. In many Asian regions, including Taiwan, OPSCC arises in a mixed etiologic environment characterized by lower HPV prevalence and substantial exposure to alcohol, betel nut, and tobacco [10,11,12,13,14]. In such populations, p16-positive OPSCC may include biologically heterogeneous tumors arising through both HPV-related and HPV-independent pathways, highlighting the need to consider regional disease patterns when applying risk stratification models developed in predominantly Western cohorts.

Recent guideline recommendations continue to support p16 immunohistochemistry as the primary initial test for identifying HPV-associated OPSCC in routine pathology practice, while recognizing that HPV-specific testing may be appropriate in selected situations, such as equivocal p16 staining patterns or discordance between p16 expression and clinicopathologic features [17]. Our findings support this pragmatic approach. Although HPV RNA ISH provides biologically specific information, it requires specialized laboratory resources and may increase diagnostic cost and turnaround time. Therefore, our results do not support universal HPV RNA testing for all OPSCC cases or treatment modification based solely on HPV RNA status. Rather, HPV RNA ISH may be most clinically useful when selectively applied to p16-positive tumors with atypical clinicopathologic features, including prominent keratinization, non–lymphoepithelial tumor location, substantial carcinogen exposure, or equivocal p16 staining. This targeted strategy preserves p16 immunohistochemistry as a practical initial screening test while using RNA ISH to refine biological characterization in cases where p16 alone may be less reliable. Future studies should validate this selective testing approach in larger, multicenter cohorts and evaluate its feasibility in real-world diagnostic workflows. In particular, prospective studies incorporating HPV RNA testing in both p16-positive and selected p16-negative tumors, additional biomarkers such as Rb expression, and formal assessment of cost, turnaround time, and clinical decision impact would help determine how best to integrate RNA-based HPV testing into routine practice.

Several limitations should be acknowledged. First, the retrospective single-center design may limit the generalizability of the findings. Second, the p16-positive/HPV RNA-negative subgroup included only five cases, and the limited number of outcome events restricted the statistical power for subgroup-specific survival analyses and multivariable modeling. Third, HPV RNA ISH was performed only in p16-positive tumors; therefore, rare p16-negative/HPV RNA-positive cases could not be identified, limiting conclusions regarding the overall concordance between p16 immunohistochemistry and transcriptionally active HPV. Fourth, carcinogen exposure data were obtained from medical records, and detailed quantitative information regarding alcohol consumption and betel nut chewing was not consistently available. Fifth, patients with follow-up shorter than six months were excluded from survival analyses to reduce bias from inadequate follow-up, but a formal comparison between excluded and included patients was not performed because of the small number of excluded cases. Sixth, we did not perform a formal cost-effectiveness or turnaround-time analysis for HPV RNA ISH. Finally, Rb immunohistochemistry was not evaluated, which limits mechanistic interpretation of HPV-independent p16 overexpression.

## 5. Conclusions

p16 immunohistochemistry remains a practical initial marker for HPV-associated OPSCC in routine diagnostic practice. In carcinogen-endemic populations, selective HPV RNA testing may be useful for p16-positive tumors with atypical clinicopathologic features, including prominent keratinization, non-lymphoepithelial tumor location, or substantial carcinogen exposure. This approach may improve biological characterization while preserving the practicality of p16-based initial screening.

## Figures and Tables

**Table 1:** Clinicopathologic comparisons between p16-positive and p16-negative oropharyngeal squamous cell carcinoma.

	Overall (n = 140)	p16+ (n = 49)	p16− (n = 91)	*p* Value
Age, y (range)	57 (35–90)	59 (38–77)	57 (35–90)	0.24
Gender				<0.01 **
Male	124 (89%)	37 (76%)	87 (96%)	
Female	16 (11%)	12 (24%)	4 (4%)	
Primary tumor site				<0.01 **
LE organ ^§^	64 (46%)	42 (86%)	22 (24%)	
Non-LE organ ^§^	74 (53%)	7 (14%)	67 (74%)	
Multifocal	2 (1%)	0 (0%)	2 (2%)	
TNM stage				<0.01 **
III + IV	78 (56%)	6 (12%)	72 (79%)	
I + II	62 (44%)	43 (88%)	19 (21%)	
T classification				0.03 *
T3, T4	42 (30%)	9 (18%)	33 (36%)	
T1, T2	98 (70%)	40 (82%)	58 (64%)	
N classification				0.43
N1–N3	103 (74%)	38 (78%)	65 (71%)	
N0	37 (26%)	11 (22%)	26 (29%)	
Carcinogen				
Alcohol	101 (72%)	25 (51%)	76 (84%)	<0.01 **
Betel nut	78 (56%)	16 (33%)	62 (68%)	<0.01 **
Cigarette	110 (79%)	29 (59%)	81 (89%)	<0.01 **
>10 pack-years	106 (76%)	26 (53%)	80 (88%)	<0.01 **
Keratinization			1	<0.01 **
Non-keratinizing	37 (26%)	32 (65%)	5 (6%)	
NK-M †	27 (19%)	13 (27%)	14 (15%)	
Keratinizing	76 (54%)	4 (8%)	72 (79%)	
Follow-up, m (range)	35.6 (0–82.0)	44.6 (0 –82.0)	30.8 (0–82.0)	

^§^. LE, lymphoepithelial; defined as lymphoepithelial-rich oropharyngeal sites, including tonsils and base of tongue in this cohort. †. NK-M, non-keratinizing with maturation. * *p* < 0.05, ** *p* < 0.01.

**Table 2:** Clinicopathologic comparisons between p16-positive/HPV RNA-positive and p16-positive/HPV RNA-negative oropharyngeal squamous cell carcinoma.

	HPV RNA-Positive (n = 44)	HPV RNA-Negative (n = 5)	*p* Value
Age, y (range)	60 (40–77)	51 (38–61)	0.08
Gender			0.18
Male	32 (73%)	5 (100%)	
Female	12 (27%)	0	
Primary tumor site			<0.01 *
LE organ ^§^	41 (93%)	1 (20%)	
Non-LE organ ^§^	3 (7%)	4 (80%)	
Multifocal	0	0	
TNM stage			0.38
III + IV	6 (14%)	0	
I + II	38 (86%)	5 (100%)	
T classification			0.92
T3, T4	8 (18%)	1 (20%)	
T1, T2	36 (82%)	4 (80%)	
N classification			0.04 *
N1–N3	36 (82%)	2 (40%)	
N0	8 (18%)	3 (60%)	
Carcinogen			
Alcohol	20 (46%)	5 (100%)	0.02 *
Betel nut	12 (28%)	4 (80%)	0.02 *
Cigarette	24 (55%)	5 (100%)	0.05 *
>10 pack-years	21 (48%)	5 (100%)	0.03 *
Keratinization			<0.01 **
Non-keratinizing	31 (71%)	1 (20%)	
NK-M †	12 (27%)	1 (20%)	
Keratinizing	1 (2%)	3 (60%)	
Follow-up, m (range)	45.5 (0.4–82.0)	36.9 (16.4–60)	

^§^. LE, lymphoepithelial; defined as lymphoepithelial-rich oropharyngeal sites, including tonsils and base of tongue in this cohort. Comparisons involving the HPV RNA-negative subgroup are exploratory because of the small number of cases. †. NK-M, non-keratinizing with maturation. * *p* < 0.05, ** *p* < 0.01.

**Table 3:** Univariable analysis for overall survival and disease-free survival.

	Overall Survival			Disease-Free Survival		
	HR	95% CI	*p* Value	HR	95% CI	*p* Value
p 16 status						
Negative	Reference			Reference		
Positive	0.16	0.05–0.52	<0.01 **	0.09	0.02–0.36	<0.01 **
Keratinization						
NK	Reference			Reference		
NK-M †	2.01	0.45–8.99	0.36	1.62	0.41–6.50	0.49
Keratinizing	4.83	1.44–16.09	0.01 *	4.29	1.49–12.40	<0.01 **
Age	0.98	0.94–1.03	0.46	0.98	0.95–1.02	0.44
Gender						
Female	Reference			Reference		
Male	INF	INF-INF	<0.01 **	INF	INF-INF	<0.01 **
Tumor site						
Non-LE organ	Reference			Reference		
LE organ	0.45	0.22–0.93	0.03 *	0.54	0.21–1.04	0.06
Clinical stage						
I–II	Reference			Reference		
III–IV	5.30	2.03–13.87	<0.01 **	6.40	2.47–16.61	<0.01 **
T stage						
T1-2	Reference			Reference		
T3-4	2.53	1.23–5.23	0.01 *	2.50	1.25–5.01	<0.01 **
N stage						
N0	Reference			Reference		
N1-3	1.41	0.58–3.44	0.45	1.71	0.71–4.15	0.23
Carcinogen						
Alcohol	9.58	1.31–70.36	0.03 *	2.38	0.83–6.76	0.10
Betel nut	3.03	1.24–7.41	0.01 *	1.78	0.85–3.75	0.13
Smoking	INF	INF-INF	<0.01 **	2.64	0.80–8.65	0.11
>10 pack-year	INF	INF-INF	<0.01 **	3.32	1.01–10.89	0.05 *

Cox proportional hazard regression. * *p* < 0.05, ** *p* < 0.01. †. NK-M, non-keratinizing with maturation. INF indicates infinite hazard ratio, reflecting complete separation or very small numbers of events in selected categories. These estimates should be interpreted cautiously.

**Table 4:** Multivariable analysis for overall survival and disease-free survival.

	Overall Survival			Disease-Free Survival		
	HR	95% CI	*p* Value	HR	95% CI	*p* Value
Age, per year	0.99	0.93–1.05	0.72	0.99	0.94–1.05	0.80
p16-positive vs. p16-negative	1.29	0.24–6.96	0.76	0.33	0.06–1.88	0.21
Clinical stage III–IV vs. I–II	11.72	1.32–103.94	0.02	8.03	1.64–39.31	0.01
Keratinizing vs. non-keratinizing	1.72	0.53–5.56	0.36	0.98	0.38–1.85	0.96
Betel nut chewing vs. no betel nut chewing	3.12	0.67–14.47	0.14	0.76	0.31–1.85	0.54

Multivariable Cox proportional hazards regression was performed using age, p16 status, clinical stage, keratinizing morphology, and betel nut chewing. Clinical stage was selected instead of individual T and N classifications to reduce collinearity. Variables with complete separation or unstable estimates in univariable analysis were not included in the main model. Results should be interpreted cautiously because of the limited number of outcome events.

## Data Availability

The de-identified data that support the findings of this study are available from the corresponding author upon reasonable request. Access to the data may require approval by the Institutional Review Board and/or a data use agreement, in accordance with institutional policies and applicable regulations. During the preparation of this work, the authors used ChatGPT (OpenAI, GPT-5.5 Thinking, accessed 15 July 2026) only to improve language readability, grammar, and manuscript clarity. The authors reviewed and edited all AI-assisted text and take full responsibility for the content of the publication.
